# Virtual immediate feedback with POCUS in Belize

**DOI:** 10.3389/fdgth.2023.1268905

**Published:** 2023-11-01

**Authors:** Anita Mulye, Ajay Bhasin, Bonita Borger, Colleen Fant

**Affiliations:** ^1^ Division of Hospital Medicine, Department of Medicine, Northwestern University Feinberg School of Medicine, Chicago, IL, United States; ^2^ Department of Family Medicine, Hillside Healthcare International Clinic, Punta Gorda, Belize; ^3^ Division of Emergency Medicine, Department of Pediatrics, Ann and Robert H. Lurie Children's Hospital of Chicago, Chicago, IL, United States

**Keywords:** POCUS, global health, LMIC, training, virtual feedback, medical education

## Abstract

Point of care ultrasound (POCUS) is a portable and accessible tool that has immense potential in low- and middle-income countries (LMIC) for diagnostic accuracy and medical education. We implemented a hybrid in-person and virtual training curriculum to teach providers in Belize the basic techniques of lung ultrasound in the diagnosis of pneumonia. Between August 2021 and June 2022, a total of eleven lung scans were performed at Hillside Clinic for patients presenting with respiratory complaints. Deidentified images were shared via the ButterflyIQ web platform to POCUS experts in the United States. We found that training was solidified through virtual, immediate feedback using the common interfaces Butterfly iQ + and WhatsApp to share images and guide diagnostic reasoning. The aim of this review study is to share our experience and challenges in the implementation of a POCUS training curriculum in an LMIC, provide an example of training methodology that can be effective, and discuss how this can be implemented and modified for clinicians in similar settings.

## Introduction

Point of care ultrasound (POCUS) is becoming a widely useful tool in low- and middle-income countries (LMIC) due to its portable nature, trainable interface and readily available data to guide clinical decision-making. POCUS education in the global health setting is also built around the goal of self-sustainable efforts for the receiving institutions (1). There have been a variety of curricula developed to train providers in the use of ultrasound in the context of global health work (2). Most training programs include didactic sessions followed by hands-on practice and/or simulation (3, 4). However, barriers and challenges exist which prevent the more widespread use of ultrasound in LMIC, including insufficient training, access to equipment and maintenance, and lack of trainee feedback (5).

Tele-ultrasound has been employed in several regions of the world as a method of providing further training and feedback to trainees (6). However, a major challenge is internet connectivity; thus, other modalities such as apps via cell phones have also been implemented (7). Using web-based applications, such as WhatsApp(™), may provide a lower cost alternative to providing real-time feedback and education sustainably. Our aim in this study was to develop a concise training curriculum using both remote and on-site modalities to introduce POCUS in a primary care clinic in Belize, as well as provide immediate feedback on technique and diagnostic accuracy via a virtual interface.

## Materials and methods

### Setting

We provided a handheld Butterfly iQ + (™) ultrasound device to a primary care clinic through a collaboration with Northwestern University and Hillside Healthcare International Clinic. The Butterfly iQ + is a handheld device measuring 163 × 56 × 35 mm and weighing 309 grams, has a battery life of at least two hours, and is fully charged in five hours. It is compatible with both iOS and Android systems via cable and thus images can be uploaded and saved through the Butterfly iQ + app on a number of devices. The decision to use this particular device was made due to the availability of tele-guidance, customer support, and a global health program which waived membership fees, making it a cost-effective choice for our introductory purposes.

Hillside Healthcare International Clinic is a free primary care clinic in Punta Gorda, Belize. It is staffed by a medical director, volunteer physicians, trainees, as well as Belizean intake staff, a lab technician and pharmacist. Hillside has a total of 2,297 patients and saw approximately 2,777 patients in 2022. There is stable internet connection at the clinic, reliable communication with phone applications, and similar time zones.

### Intervention

We provided a two-day training session totaling eight hours on lung POCUS on-site for the medical director (a Family Medicine provider), intake staff, lab technician and resident and student volunteers. This structure was adapted from prior curriculum implemented in Tanzania, and was modified to fit the needs and time constraints of the intended learners (Supplementary Appendix S1). This consisted of a one-hour didactic session followed by hands-on practice with a POCUS expert. A pre- and post-test of trainees' understanding of lung ultrasound was conducted before and after the session (Supplementary Appendix S2). Subsequently, three additional internal medicine physicians were trained at Northwestern University prior to their departure to Hillside as faculty volunteers. Images were uploaded via the Butterfly iQ + app so instructors in the US could provide feedback on images gathered by trainees and staff. We created a WhatsApp(™) group to notify instructors of new images and videos in order to provide immediate feedback on quality and interpretation.

Trainees acquired images on-site during clinical encounters over six months following the initial training. During the image acquisition phase, the on site scanner would alert a cohort of POCUS experts via WhatsApp (™) to provide either live image review or image review within 24 h. On average, the time to respond from the U.S. POCUS experts was between 10 and 60 min. Deidentified images were shared via the ButterflyIQ web platform. When relevant, clinical background was also shared to help guide diagnostic reasoning. In one case, the device feature of live guidance was utilized to guide technique and image acquisition in real-time during the patient encounter.

## Results

Between August 2021 and June 2022, a total of eleven lung scans were performed at Hillside Clinic for patients presenting with respiratory complaints (Table 1). All scans were reviewed and directed feedback provided within 24 h and eight out of eleven were reviewed by remote POCUS experts during the clinical encounters.

**Table 1:** Results and clinical findings from POCUS scans.

Age	Chief Complaint	POCUS findings	Final Diagnosis	Antibiotics prescribed?	Notes
17 mo	Fever	A lines, no infiltrate	Viral	No	Exam: wheezing; treated with amoxicillin 2 weeks with initial improvement then fever
67 yo	6 months cough	A lines, no infiltrate	Asthma	No	No fever, dyspnea, smoking, history of allergies
6 yo	1 day cough, congestion	A lines, no infiltrate	Viral	No	History of asthma, allergies
9 yo	2 days wheezing, asthma	A lines, no infiltrate	Asthma, URI	No	No fever
4 yo	1 month cough	A lines, no infiltrate	Unclear	No	
4 yo	2 days cough and wheezing, subjective fever	A lines, no infiltrate	Asthma, URI	No	Afebrile in clinic, monthly visits for similar presentations, improved with nebulizers and steroids.
44yo	1 week cough, crackles on exam	B lines	PNA	Yes	Can improve gain by sliding to left
6 yo	4 weeks cough, afebrile, possible hemoptysis	A lines, no infiltrate		No	Gain too high so difficult to see A lines as well
4yo	Cough, wheezing	B lines, Left lobe	PNA	Yes	
8 yo	Cough, fever, wheezing on exam	B lines, Right lobe	PNA	Yes	
51 yo	Chest pain, dyspnea, wheezing and crackles on exam	B lines	PNA	Yes	

URI, upper respiratory illness; PNA, pneumonia; A lines, normal lung finding; B lines, abnormal lung finding, could indicate pneumonia or fluid.

Eight patients scanned were pediatric patients and three were adults. All presented with respiratory symptoms such as cough, shortness of breath, chest pain and fever. All images were deemed adequate to inform care decisions and only those with visible B-Lines (an ultrasound sign that can indicate a consolidation or pneumonia in the right clinical setting) were prescribed antibiotics. Four patients received antibiotics based on the evidence of B-lines. Clinical staff reported that a POCUS scan that did not indicate pneumonia influenced care decisions such that those patients did not receive antibiotics when they otherwise would have. Per direct provider interviews, in patients where the clinical syndrome was unclear, “seeing B-lines on ultrasound pushed me to prescribed antibiotics when I was 50/50.”

## Discussion

Our intervention demonstrates POCUS can improve access to care to patients in LMIC by leveraging novel technologies and devices. Moreover, through these technologic interfaces, improved collaboration between those in resource-rich and LMIC countries can facilitate knowledge transfer and care delivery.

Hillside clinic does not have access to x-rays. Prior to this intervention, any patient requiring an x-ray would be referred to the local hospital which would require transport and may result in a cost that most patients could not afford, or receive empiric treatment without x-ray results. Our intervention utilizing cross-national and cross-institutional POCUS improved care by allowing local clinicians to assess for pneumonia in real-time, more accurately prescribe antibiotics, and more appropriately allocate resources. Though uptake of the technology had a clear lag phase as staff gained comfort in technique, over time, use increased and image quality and interpretation improved per expert assessment.

The use of virtual immediate connection with POCUS experts added another layer of support and improvement in diagnostic accuracy, as well as continued learning for trainees through feedback.

One limitation of this project was the lower number of patients than anticipated who presented with respiratory complaints and may have benefited from an x-ray. Due to the COVID-19 pandemic, the Ministry of Health in Belize had initially restricted patients with respiratory symptoms from general primary care settings, and instead directed them to the local hospital for COVID-19 testing and treatment. This limited the degree of practice one could obtain in lung POCUS in a given timeframe. Given numerical limitations, while qualitative perspectives of treatment practices could be assessed, no change in practice could be measured statistically.

While it was intended, it was difficult to follow up with patients after the encounter regarding their symptoms, particularly if they were not prescribed antibiotics. This was due to the lack of designated staff to be able to carry out this task on a routine basis. One way this could be rectified in the future is through delegation to one of the student volunteers to call the patient or set up a follow up visit within a certain time period. However, this would also require additional repeat training each time a new short-term volunteer was tasked with this.

For the majority of the study period, there was one provider who performed the ultrasounds and obtained virtual feedback. This was mainly due to clinic workflow and feasibility of multiple providers using the device during the four-hour timeframe during which the clinic operates. Given we trained volunteer providers prior to their departure as well, in the future we aim to have multiple providers using the device and providing a range of data to monitor the progress of training and utility of the device across several learners.

## Conclusion

POCUS is a diagnostic tool that is becoming more widely used in low- and middle-income settings. New technological innovations provide opportunities to educate and collaborate remotely and virtually. In the future, we aim to broaden the scope of this project by including different training exams (heart, skin and soft tissue, and abdominal, for example) and train additional clinical staff to ensure sustainability of implementation so that the clinic and community will be able to derive long-term benefit from our efforts. From our findings in this hybrid in-person and virtual training to providers in Belize, the application of real-time image interpretation and feedback provided learners an accessible and user-friendly interface to communicate with POCUS experts. The ultimate aim was to instill competency in the use of POCUS in a limited time frame by supporting novices in the early learning phases. The impact of this intervention in LMIC includes the introduction of technology to a resource-scarce region in a controlled and guided manner with additional support to ensure competency. In the future, we hope to continue with the use of the Butterfly and WhatsApp applications to maintain a continuous stream of communication with the clinic. We also hope that with gaining competency, the trained staff will in turn train oncoming providers and staff to ensure that there is at least one person skilled in POCUS at any time in the clinic. A third goal for the future is the eventual training of other areas of focus, such as cardiac, abdominal, skin and soft tissue ultrasound.

## Data Availability

The original contributions presented in the study are included in the article/Supplementary Material, further inquiries can be directed to the corresponding author.
