# Supplementary material for: Virtual immediate feedback with POCUS in Belize
Source: Front Digit Health. 2023 Nov 1;5:1268905. doi: 10.3389/fdgth.2023.1268905 (PMC10649964; doi:10.3389/fdgth.2023.1268905)
Supplement: Supplementary file 1 [file Table1.pdf]

Appendix 1: POCUS Training Curriculum

Day	Content	Supplies
One	Didactic Portion: Basics of Lung Ultrasound (60 minutes)<br><br>Phantom Practice: Scanning a Lung Model (30-60 minutes)<br><br>Deliberate Practice: Scanning a Standardized Patient (30-60 minutes)	Powerpoint presentation<br><br>Lung Phantom (pork rib, sponge, tray, water)<br><br>Ultrasound machine (ButterflyIQ)<br><br>Ultrasound Gel<br><br>Lung Ultrasound Checklist
Two	Deliberate Practice: Scanning a Standardized patient (30-60 minutes)	Ultrasound Machine (ButterflyIQ)<br><br>Ultrasound Gel<br><br>Lung Ultrasound Checklist
Three-Five	In-Situ Scanning: Scanning available patients in clinic and on wards	Ultrasound Machine (ButterflyIQ)<br><br>Ultrasound Gel<br><br>Lung Ultrasound Checklist
