# Supplementary material for: Virtual immediate feedback with POCUS in Belize
Source: Front Digit Health. 2023 Nov 1;5:1268905. doi: 10.3389/fdgth.2023.1268905 (PMC10649964; doi:10.3389/fdgth.2023.1268905)
Supplement: Supplementary file 2 [file Table2.pdf]

Appendix 2: Lung Ultrasound Checklist

	**You have a patient in front of you who is being evaluated for pneumonia. Please perform a complete Lung Ultrasound exam.** ***(Can perform study with patient supine, elevated HOB or left lateral decubitus)***	Done correctly	Partially Done/Done Incorrectly	Not Done
1	Uses Pediatric (<6) or Adult Lung preset to assess pleura, and parenchyma with Butterfly device			
2	Places probe longitudinally so that probe marker = cephalad			
3	Adjusts gain appropriately so al structures are visible<br><br>Utilized Lung Sweep approach<br>Performs anterior, lateral and posterior chest sweeps on both sides until the solid organ is visualized on the inferior aspect of that sweep<br>Records and labels images			
4	Can identify pleural line and lung sliding if present			
5	Identify ribs<br><br>Identify A lines<br><br>Identify B lines where present			
6	Identify abnormal findings if present:<br><br>Lung consolidation<br>Shred Sign<br>Focal B lines<br>Air bronchograms<br>Pleural effusions<br>Attempts to identify or rule out small dependent pleural effusion			
